# Supplementary material for: Updating the Clinical Application of Blood Biomarkers and Their Algorithms in the Diagnosis and Surveillance of Hepatocellular Carcinoma: A Critical Review
Source: Int J Mol Sci. 2023 Feb 21;24(5):4286. doi: 10.3390/ijms24054286 (PMC10001986; doi:10.3390/ijms24054286)
Supplement: Supplementary file 1 [file ijms-24-04286-s001.zip › ijms-2219229-supplementary.pdf]

Supplementary Material

1 Biomarkers needing further validation

Figure S1 depicts the timeline and number of articles published on HCC biomarkers needing further validation up to 2023.

Figure S1: Timeline and number of articles (in parenthesis) published on hepatocellular carcinoma biomarkers that need further studies up to 2023.

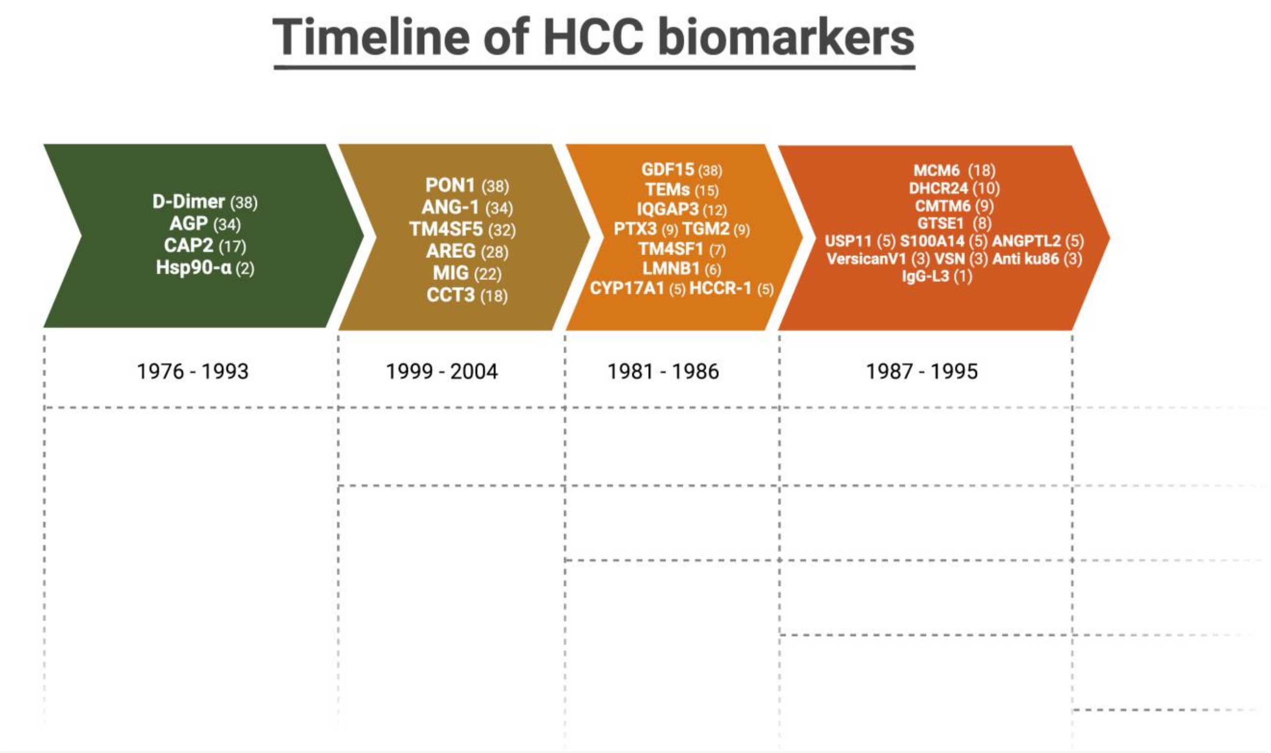

The following HCC biomarkers require additional validation (**Table S1**):

Table S1: Example of HCC diagnostic biomarkers requiring further validation. Sensitivity, specificity and AUROC shown as percentages (%).

Biomarker	Type	Study Type	Sensitivity (%)	Specificity (%)	AUROC (%)	Cut-off	Ref.	Notes
GDF15	Immunological	Retrospective	63.1	86.6	84.2	1.94 ng/mL	*Liu., X et al., 2015*	Cirrhosis any etiology, CLD
CAP2	Protein	Retrospective	78.6-82.6 (Early vs late HCC)	79.3-81.4 (Early vs late HCC)	86	8.24 ng/mL	*Chen, M., et al, 2015*	Cirrhosis any etiology, early and late HCC, CLD
IQGAP3	Protein	Retrospective	74.5-81.6 (α-FP-/early HCC)	71.6	75	43.5 pg/mL	*Qian, E.N., et al, 2016*	Cirrhosis any etiology, CLD
Fucosylated glycoproteins	Protein	Retrospective	61.2	81.6	73	-	*Shang S., et al, 2016*	Cirrhosis any etiology
OPN	Protein	Metanalysis	81	87	-	-	*Sun, T. et al., 2018*	CHB
Vimentin	Protein	Prospective	96	65	-	-	*Idris N.K., et al, 2019*	CLD any etiology and cirrhotics
D-Dimer	Protein	Metanalysis	75	93	88	-	*Fang P., et al, 2020*	CLD any etiology and cirrhotics
PON1	Protein	Retrospective	70.7	78.1	75	191.1 ng/mL	*Ding, G., et al, 2020*	Cirrhosis (HCV and HBV prevalent), CLD

Pentraxin-3	Immunological	Retrospective	-	-	93 (CHB) 92 (Early HCC) 95 (α-FP-)	9.23 ng/mL (CHB) 9.35 ng/mL (Early HCC) 8.98 ng/mL (α-FP-)	*Deng H et al., 2020*	Cirrhosis any etiology, CLD, early HCC, CHB without HCC
FGF-19	Protein	Retrospective	100	90	98	>180 pg/mL	*Mohamed G.A., 2022*	Cirrhosis

1.1 Protein Biomarkers

1.1.1 PON1

Paraoxonase 1 (PON1) was found to have the potential to be effective biomarkers for distinguishing α-FP -negative HCC from cirrhosis in a retrospective 2021 study(1).

The AUROC for PON1 in differentiating early HCC from liver cirrhosis has been found to be 89% in a 2012 study, with a sensitivity of 71.4% and a specificity of 94.7%. The findings suggested that glycan differences in serum PON1 could be used as potential glycan biomarkers for distinguishing early HCC from patients with liver cirrhosis(2,3).

Variations in PON1 glycosylation may be linked to α-FP -negative HCC and could be used as potential glycomic-based biomarkers to differentiate α-FP-negative HCC from cirrhosis(4). Indeed, Fuc-PON1 (the ratio of fucosylated serum paraoxonase 1 to total serum paraoxonase 1) was found to be significantly higher in HCC patients with low α-FP levels. To validate Fuc-diagnostic PON1's potential, a separate cohort of α-FP-negative early HCC patients was studied. Fuc-PON1 had an AUROC of 78%, sensitivity of 62.2%, specificity of 67.7%, and accuracy of 64.5%. The current study's findings confirmed the clinical utility of Fuc-PON1, demonstrating its superior diagnostic potential for distinguishing α-FP negative early HCC from liver cirrhosis patients(5).

Serum PON1 could be used to detect microvascular invasion. The optimum diagnostic cut-off value for PON1 was determined by ROC curves to be 191.12 ng/mL (AUROC 75.4%, sensitivity 70.67%, specificity 78.11% in the test cohort), which was significantly better than α-FP (cut-off 279.8 ng/mL, AUROC 66.6%, sensitivity 40.38%, specificity 85.19%, P = 0.0063). The validation cohort corroborated these findings(6).

1.1.2 CAP2

Cyclase-associated protein 2 (CAP2) is a biomarker for HCC patients that may be especially useful for early-stage HCC detection and when plasma α-FP levels are negative. In particular, the findings of a preliminary study involving 86 HCC patients, 59 cirrhotic patients, and 30 healthy people revealed that CAP2 and α-FP plasma levels in HCC patients were significantly higher when compared to cirrhosis and controls. CAP2 had higher sensitivity than α-FP (82.6% vs 59.3%) for general HCC patients and early-stage HCC patients (78.6%

vs 40.4%). Furthermore, CAP2 can predict 82.9% of HCC in patients who do not have α-FP(7). The CAP2 protein has been found to be overexpressed in hepatocellular carcinoma (HCC), human breast cancer, and malignant melanoma(8). It has been reported that CAP2, could serve as molecular markers for early HCC(9).

CAP2 is elevated in HCC and is a poor prognostic biomarker for patients with this lethal disease. The prognostic significance of CAP2 in HCC was confirmed further in a validation cohort of 208 HCC patients and through stratified survival analysis(10).

1.1.3 IQGAP3

IQ motif contains GTPase-activating proteins (IQGAPs) are a type of scaffolding protein that regulates a variety of cellular activities by facilitating cytoskeletal remodelling and signal transduction(11). The IQGAP3 is widely overexpressed in several human cancers, including those of the liver, ovary, lung, large intestine, gastric, bone marrow, and breast(12).

A 2017 study discovered that IQGAP3 functions as an important regulator of metastasis and epithelial-to-mesenchymal transition in HCC by constitutively activating the TGF-Beta signalling pathway, providing new evidence of IQGAP3's role in metastasis and indicating its potential as a prognostic biomarker candidate(12). In HCC patients, a high level of IQGAP3 predicted poor survival(13).

IQGAP3 levels in HCC patients' plasma were significantly higher than in cirrhotic patients and healthy controls ($P < 0.01$)(14). Furthermore, IQGAP3 proteins could detect α-FP -negative HCC patients with sensitivity and specificity of 70.5% and 71.6%, respectively(14). IQGAP3 sensitivity was 74.4% for the detection of small HCCs.

1.1.4 Vimentin

Vimentin has been found to be a promising biomarker for detection of early HCC(15). Vimentin, which is dependent on the SH3 domain protein 1 (LASP1), is required for HBX-mediated epithelial-mesenchymal transition and hepatocarcinogenesis(16).

High S100A4 and vimentin expression and low E-cad expression correlate with an aggressive, malignant phenotype in HCC. These results also support a role for E-cad as a prognostic factor in HCC(17).

Anti-Ku86, lamin B1, and vimentin levels in the blood could be used as surrogate markers for HCC, either alone or in combination with α-FP. HCC patients had higher levels of vimentin mRNA than CLD patients and controls (sensitivity, 94%; specificity, 92%), and ROC curves for differentiating HCC from liver cirrhosis revealed a higher AUROC for vimentin than for α-FP, lamin B1, and anti-Ku86 for the diagnosis of HCC ($P < 0.001$)(18).

1.1.5 D-Dimer

Ascites and HCC are the main factors associated with increased fibrinolytic activity in patients with liver cirrhosis(19).

Fibrinogen and D-dimer levels, which rise after carcinogenesis, may be simple but effective predictors of poor tumour profiles and outcomes in HCC(20).

One of the severe complications of HCC that influences the prognosis of patients with liver cirrhosis and HCC is portal vein thrombosis. As a result, D-dimer testing has been proposed as a sensitive marker for the diagnosis and prognosis of HCC patients with portal vein thrombosis. Indeed, D-dimer levels in 118 HCC patients with portal vein thrombosis were significantly higher than in HCC patients without portal vein thrombosis, $P < 0.002$(21).

The pooled sensitivity, specificity, positive and negative likelihood ratios, and DOR of plasma D-dimer levels for the diagnosis of HCC in a 2020 Chinese meta-analysis were 0.75, 0.93, 11.4, 0.27, and 42, respectively, and the AUROC was 88%(22).

re, D-dimer could be a useful biomarker for the detection of HBV-related HCC. The combined detection of DCP, α-FP, and D-dimer had a higher diagnostic value for different types of HCC than individual biomarker detection(23).

1.1.6 Fucosylated glycoproteins

N-linked glycosylation changes are known to occur during cancer development. Fucosylation of serum glycoproteins was found to be significantly higher in HCC compared to liver cirrhosis(24).

The usefulness of some of these proteins in the diagnosis of HCC was determined using a high-throughput plate-based approach on over 300 patient samples. The best results were obtained with fucosylated hemopexin, which had an AUROC of 95% and an optimal sensitivity and specificity of 92%(25).

Fucosylated kininogen and fucosylated alpha-1-antitrypsin levels were significantly higher in HCC patients compared to cirrhotic patients ($P < 0.0001$) (26).

Aberrant glycosylation of target glycoproteins is a common and important event in many cancers, such as increased fucosylated haptoglobin (Hp) in HCC (27,28). In distinguishing HCC from healthy controls, it had an AUROC of 73%, sensitivity of 61.22%, specificity of 81.63%, and accuracy of 71.43%. As a result, Fuc-Hp may be useful as a glycobiomarker in the diagnosis and prediction of HCC(27). Similarly, the elevated bifucosylation degree of haptoglobin can distinguish early-stage HCC patients from cirrhosis in each etiologic category, which could be used as a potential marker for early detection and prediction of HCC in cirrhotic patients(29).

Fuc-hemopexin (HPX), in particular, is a valuable biomarker for HCC, but it may be a marker for hypercarcinogenic liver rather than tumour-bearing liver(30). However, in Japanese HCC patients Fuc-HPX had lower sensitivity and specificity when compared to other markers like GP73, α-FP, and DCP (AUROC 72% vs 81%, and 90%, respectively)(31).

Fucosylated haptoglobin (Fuc-Hpt) is a novel and potentially useful biomarker for predicting liver disease progression and HCC development(32).

Also, other authors discovered in 2016 that alpha-1-acid glycoprotein with multifucosylated tetraantennary N-glycans was significantly elevated in HCC patients but not the single fucosylated derivative(33).

Furthermore, a 2020 study suggests that serum glycoprotein defucosylation may occur during the development and progression of HCC(34).

Recently, some researchers discovered that the ratio of fucosylation of a tri-antennary glycopeptide from site N762 increased significantly from cirrhosis to early HCC ($P = 0.0486$). This fucosylation ratio of a tri-antennary glycopeptide could be a promising candidate for early detection of NASH HCC and should be validated in a larger sample set(28).

1.1.7FGF

Fibroblast growth factors (FGFs) are a large family of polypeptide cytokines that have a wide range of functions, including cell growth, angiogenesis, wound healing, and tissue repair(35).

The FGF family and their receptors (FGFRs) are involved in a wide range of biological activities, including embryonic development, proliferation, differentiation, survival, angiogenesis, and migration, among others.

It has previously been proposed that FGF is involved in the invasion of HCC into surrounding tissues(36).

FGF8, FGF17, and FGF18 are involved in autocrine and paracrine signalling in HCC, enhancing tumour cell survival under stress, malignant behaviour, and neoangiogenesis. As a result, the FGF8 subfamily promotes the development and progression of HCC(37).

The Cancer Genome Atlas data mining results showed that FGF and/or FGFR expression is high in HCC tumours compared to normal tissues.

Furthermore, substantial evidence suggests that the FGF/FGFR signalling axis is important in a variety of mechanisms that contribute to HCC development(38).

Furthermore, it has been suggested that FGF-2 levels are elevated prior to the emergence of HCC(39).

Also, FGF-19 could be a novel non-invasive marker for HCC(40). FGF 19-fibroblast growth factor receptor 4 (FGFR4) overexpression has been identified as an oncogenic driver pathway in HCC patients. As a result, the FGF19-FGFR4 signalling pathway is a promising target for HCC treatment(41). FGF19 collaborates with other signalling pathways in the development of primary liver cancer, including the EGFR, Wnt/-catenin, the endoplasmic reticulum-related signalling pathway, STAT3/IL-6, RAS, and extracellular signal-regulated protein kinase(42).

When compared to control and cirrhotic groups, the HCC group had the highest level of FGF-19 in serum ($P < 0.001$). FGF-19 correlated positively with α-FP ($r = 0.383$, $P = 0.003$). FGF-19, had an AUROC of 98%, sensitivity of 100%, specificity of 90%, PPV of 90%, NPV of 100%, and total accuracy of 98% at a cut-off point of > 180 pg/mL(43).

1.1.8–KLF4

Kruppel-like factor 4 (KLF4) is a well-known tumour suppressor found in a variety of cancers. CD9 and CD81 were discovered to be new transcriptional targets of KLF4, and dysregulated KLF4-CD9/CD81-JNK signalling was discovered to contribute to HCC development(44). In detail, loss of KLF4 expression may contribute to oncogenic TGF-Beta signalling activation and subsequent HCC progression(45). Positive KLF4 expression in HCC patients should be correlated with survival time and considered promising prognostic biomarkers(46).

1.1.9 Anti ku86

Ku86 is a nuclear protein that is involved in a variety of biological processes(47).

Anti-Ku86 levels in the blood could be used as a surrogate marker for HCC, either alone or in combination with α-FP. In a 2019 study, HCC patients had higher levels of anti-Ku86 with a sensitivity of 94% and a specificity of 80% when compared to CLD patients and control(48).

1.1.10 IgG-L3

Elevated core-fucosylated IgG was discovered in 2015 as a new diagnostic and prognostic marker in HBV-related HCC(49). In the training set and validation cohort 1, IgG-L3% had better general diagnostic performance than α-FP (accuracy: 81.33-85.11% versus 63.33-78.61%). The diagnostic accuracy of IgG-L3% was 72.54-73.60% in validation cohort 2 (α-FP-negative HCC patients). However, no other studies have been conducted.

1.1.11 EGF

Although the mechanism is unknown, epidermal growth factor (EGF) and its receptor (EGFR) play an important role in cancer proliferation and metastasis.

The potential role of heparin-binding epidermal growth factor-like growth factor (HB-EGF), a member of the EGF family, in hepatocyte neoplastic transformation has been investigated. In a previous study, the increased expression of immunoreactive HB-EGF on the cell suggested that HB-EGF may play an autocrine and/or juxtacrine role in the development or progression of human HCC(50).

A 2014 study discovered that EGF-EGFR signal pathways regulated HCC proliferation, metastasis, and inflammatory cytokine production(51).

Epidermal growth factor (EGF) overexpression causes highly malignant HCC and activated EGF/EGFR signalling is associated with an aggressive phenotype. EGF was found to be highly expressed in HCCs and to be positively associated with tumour grade. A study in particular highlighted the potential role

of EGF in promoting HCC metastasis, as well as a novel pathway for regulating fibronectin expression that could provide targets for HCC prevention and treatment(52).

Specifically, larger studies with more diverse ethnic populations are needed to confirm that the EGF rs4444903 polymorphism is a genetic contributor to liver cirrhosis and HCC in the general population(53).

The EGF + 61A/G polymorphism was found to be significantly associated with the risk of HCC in a 2022 pooled analysis(54). In Egyptian patients with chronic liver disease, the EGF 61GG genotype may be associated with an increased risk of developing HCC(55).

1.1.12 HGF

The level of hepatocyte growth factor (HGF) in the serum represents the degree of carcinogenicity in the liver of patients with CHC and cirrhosis(56). An increase in HGF could indicate disease progression and a distinction between the pathological mechanisms involved in HCC(57).

The HGF)/cMET axis is a critical signalling pathway in HCC and is strongly linked to its highly malignant characteristics(58).

The investigation of cirrhotic patients with and without HCC suggests that HGF levels may be useful for monitoring the recurrence of HCC after cirrhosis diagnosis. HGF levels in cirrhotic patients with HCC are significantly higher ($P = 0.017$) than in those without HCC(59).

In a 2014 study, 54 patients with HCC were studied. Patients with HCC had significantly higher baseline serum HGF levels than the control group ($P < 0.001$). Serum HGF, on the other hand, had no significant negative effect on survival(103).

1.1.13 VEGF

Vascular endothelial growth factor (VEGF) is a major driving force in both physiological and pathological angiogenesis, and several studies have found it to be overexpressed in HCC.

It was thought that VEGF expression in HCC tissues was related to histological grade(61).

Polymorphisms in VEGF may be important prognostic indicators for HCC patients (62).

According to a 2009 meta-analysis, tissue and serum VEGF levels appear to have a significant predictive ability for estimating overall survival in HCC and may be useful in defining prognosis in HCC(63).

Serum VEGF levels in 30 patients with liver cirrhosis were higher in the HCC group than in the cirrhosis group than in the control group. At cut off 268, the sensitivity and specificity of makers in diagnosing HCC were 60% and 92%, respectively. As a result, VEGF may be a useful serum marker for the detection of HCC(64).

Specifically, serum VEGF levels may be a useful predictor of the presence of HCC in patients with CHC(65).

In detail, in HCC patients, serum VEGF-A levels, a biological marker of angiogenesis, are an independent predictor of survival(66). Furthermore, VEGF-C expression is linked to the progression of HCC(67).

In a 2021 study involving 100 patients, those with HCC had significantly higher serum VEGF levels than the non-HCC groups ($P < 0.01$). A VEGF cut-off value of 250 pg/mL provided 80% sensitivity and 81.7% specificity for distinguishing HCC patients from non-HCC patients. In HCV patients, serum VEGF has been shown to be potentially reliable biomarkers for early and accurate HCC diagnosis(68).

In a 2022 study, 230 people with HCC, cirrhosis, or HCV and controls were tested for VEGF discriminatory power between HCC patients and controls ($AUC = 0.71$). This study confirms that the expression of the VEGF gene confers both sensitivity and specificity for HCC diagnosis(69).

1.1.14 ANG-1

Angiopoietin 1 (ANG-1) and its antagonist, angiopoietin 2 (ANG-2) are ligands that control the Tie2 receptor. The expression of ANG-1 and ANG-2 plays an important role in the angiogenic and dedifferentiation processes in HCC. In particular, the ANG-2 gene is overexpressed in hypervascular HCC(70).

Serum ANG-2 levels were significantly higher in patients with HCC than in healthy subjects and patients with cirrhosis, according to this study of 149 subjects. Serum ANG-2 levels above a certain threshold are linked to advanced HCC tumour characteristics(71).

Two previous studies found that increased Ang-2/1 expression in the presence of VEGF may play a critical role in tumour angiogenesis and progression in human HCC(72).

In a 2021 study of 240 prospectively enrolled HCC patients, plasma levels of Ang-2 correlated with liver function, tumour stage, and tumour invasiveness, outperforming α-FP, Ang-1, and VEGF in predicting survival(73).

1.1.15 TGF

Transforming growth factor-beta (TGF-β) is a multifunctional growth factor that regulates cell growth and differentiation, angiogenesis, extracellular matrix formation, immunosuppression, cancer development, and even survival. Furthermore, TGF-beta promotes HCC pathogenesis and high expression in HCC patients predicts a poor prognosis(74). Mutations and TGF-β expression patterns have been discovered to differ between tumour types.

In particular, TGF-β has the potential to be used as a biomarker for HCC. Previous research has shown that serum TGF-β levels, particularly in those infected with HCV, are linked to the development of HCC(75). Other studies have looked at TGF-β 's role in HCC, but not as a stand-alone biomarker; rather, it has been combined with the expression of other proteins or mRNA(76). A few of these studies were also conducted in single-country populations and were not validated in other contexts.

Additionally, a 2017 meta-analysis of twelve qualified articles involving 2,021 HCC patients found that those with higher TGF-1 expression had a shorter survival than those with lower TGF-1 expression ($HR = 1.42$, $P < 0.05$). Univariate analyses in the meta-analysis revealed that HCC patients with higher TGF-1 expression had a shorter survival (pooling $HR = 1.71$, $P < 0.01$) than patients with lower TGF-1 expression(77).

Despite abundant TGF-1 expression in the liver, steatosis to HCC progression causes elevated TGF-1 levels, contributing to poor prognosis and survival. TGF-1 is an important diagnostic and prognostic biomarker in HCC(78).

1.1.16 Thioredoxin

For differentiating very early HCC from non-HCC, thioredoxin had an AUROC of 0.90, sensitivity of 75.2%, and specificity of 88.9%, which were higher than that of α-FP (AUROC 0.77, sensitivity 70.1%, specificity 79.4%)(79). These findings indicate that thioredoxin has the advantage over α-FP for HCC detection, particularly for very early ANHC.

1.1.17 E2F transcription factors

E2F Transcription Factor 1 (E2F1) has been known for decades as a retinoblastoma protein (RB) binding transcription factor that regulates the cell cycle(13,80). By activating the ERK/mTOR pathway, the E2F1/USP11 signal axis promotes HCC proliferation and metastasis while inhibiting autophagy(81).

In 238 paired specimens from human HCC patients, the mRNA expression of E2F1 and ISX was highly correlated with disease pathogenesis, patient survival time, progression stage, and poor prognosis, indicating that E2F1 is an important downstream gene of ISX in HCC progression(82).

In pathological tissues derived from HCC patients, E2F1 correlates positively with IQGAP3 and both of these factors are highly expressed (R =

0.6716). In addition, a high level of E2F1 or IQGAP3 predicted poor survival in HCC patients(13). E2F1 not only transactivates cell-cycle-related factors, but it also promotes HCC proliferation by activating PKCα phosphorylation(13).

A 2021 study that looked into the role of transcription factors E2F1 and E2F2 in NAFLD-related HCC and their involvement in metabolic rewiring during disease progression found E2F1 and E2F2 transcription factors to be metabolic drivers of HCC(83).

1.1.18 GTSE1

In another 2019 study comparing GTSE1 knockdown and wild-type HCC cells, the authors discovered 979 differentially expressed genes, among which GTSE1, along with CDC20, PCNA, and MCM6, resulted in a synergistic promotion of adverse prognosis in HCC by activating the cell cycle. As a result, the latter genes could be useful prognostic molecular biomarkers in liver cancer(84).

1.1.19 USP11

USP11 (ubiquitin-specific protease 11) is a deubiquitinating enzyme that regulates multiple signalling pathways including p53, NF-B, TGF-Beta, and Hippo(85). USP11 is dysregulated in many cancers and plays a role in tumour development and progression. USP11 destabilizes Krüppel-like factor 4 (KLF4) through the removal of K63-dependent polyubiquitination, thereby inhibiting KLF4 expression(86).

A substantial body of evidence points to a link between UPS11 and tumorigenesis. It was discovered that USP11 may promote HCC cell metastasis in a pilot series of 71 HCC clinical samples, and it was provided the first evidence of the prognostic significance of USP11 expression in HCC. Furthermore, elevated USP11 and decreased KLF4 levels were found in a hepatic steatosis in vitro model as well as in clinical data from NAFLD and HCC patients(85).

The mechanism underlying USP11's role in HCC cell proliferation and metastasis was dependent on NF90, and USP11 expression in human HCC tissues was positively correlated with NF90 expression(87).

1.1.20 TGM2

A multifunctional protein, transglutaminase 2 (TGM2), may serve as a novel histological/serologic candidate involved in HCC, especially for the individuals with normal serum α-FP(88).

TGM2 is upregulated in activated hepatic stellate cells via pseudohypoxia, promoting epithelial-mesenchymal transition in HCC-derived cells both in vivo and in vitro, according to quantitative proteomics and ingenuity pathway analysis(89). TGM2 may also contribute to early HCC recurrence via signalling pathways unrelated to EMT and integrin signalling(90).

1.1.21 Vasorin

Vasorin (VSN) is a secreted cell surface protein that is associated with vascular injury repair by preventing TNF-mediated apoptosis and inhibiting TGFβ signalling(91), and its overexpression in some human tumours can promote malignant progression and angiogenesis(92). The function of VSN includes abrogation of TNF-mediated apoptosis and inhibition of TGFβ signalling but is not fully understood.

VSN promotes proliferation and migration of cells while hindering cell apoptosis in HCC, making it an attractive biological therapeutic target. When compared to the control cohorts, higher VSN levels in HCC serum were confirmed, with an AUROC of 0.77, sensitivity of 69%, and specificity of 80.5% for the diagnosis of HCC; VSN was positive in 62% of α-FP-negative HCC patients, indicating that VSN may be a potential biomarker for HCC diagnosis(93).

1.1.22 CYP17A1

The hepatic P450 enzyme cytochrome P450, family 17, subfamily A, polypeptide 1 (CYP17A1) was found to be overexpressed in the liver tissues of transgenic mice at both preneoplastic and neoplastic stages. CYP17A1 mRNA and protein levels were found to be significantly higher in human HCC tissues after mouse-to-human validation. Immunohistochemical studies revealed that CYP17A1 was overexpressed in 67% of HCC, with strong staining in well-differentiated HCCs. As a result, CYP17A1 is overexpressed in HCCs and has great promise as a non-invasive marker for HCC detection(94).

1.1.23 CMTM6

Through its interaction with and stabilization of vimentin, CMTM6 plays an important role in HCC proliferation, migration, and invasion. CMTM6 could be a potential biomarker and therapeutic target for HCC(95).

Downregulation of CMTM6 is linked to HCC metastasis and patient prognosis(96).

1.1.24 AGP

Alpha-1 acid glycoprotein (AGP) could be used as a marker for early detection of HCC in various aetiologies. AGP's sialylation and fucosylation changes could be used as a serum biomarker for HCC and cirrhosis(97).

A distinct trifucosylated tetra-antennary glycan was found in HCCs. ROC curve analysis revealed that the trifucosylated N-glycan of AGP could distinguish HCC from cirrhosis with AUC values of 0.71, 0.73, and 0.75 for NASH, ALD, and HCV, respectively(98). Furthermore, AGP could be used as a marker to monitor α-FP-negative HCC patients.

1.1.25 HCCR-1

It has been suggested that Human cervical cancer oncogene (HCCR-1) may be used in addition to α-FP to detect HCC. The sensitivities of HCCR-1 (10 ng/mL) in HCC were 44.2%, in a prospective study of 120 normal and 524 CLD patients(99). Therefore, HCCR-1 may be a useful biomarker for HCC, and the diagnostic rate may be significantly improved when HCCR-1 and α-FP are used together.

1.1.26 S100A14

Breast Cancer Membrane Protein 84 (S100A14) has been linked to the progression of several cancers and it has been suggested to be a potential HCC prognostic marker and therapeutic target(100).

S100A11 oncogenic factor overexpression promotes inflammation/fibrosis in vivo and is significantly associated with high-grade HCC with a poor prognosis(101).

However, few studies have been conducted to confirm the initial hypotheses and a potential diagnostic role in HCC.

1.1.27 ISX

The intestine-specific homeobox (ISX) proto-oncogene, is involved in cell proliferation and progression of HCC(82,102). PCAF, BRD4, and ISX mRNA expression in 377 paired specimens from 377 patients with HCC and adjacent normal tissues showed a tumour-specific expression pattern that was highly correlated with disease pathogenesis, patient survival time, progression stage, and poor prognosis(103).

1.1.28 LMNB1

Lamin B1 (LMNB1) is a clinically useful biomarker for early stages of HCC in tumour tissues and plasma.

In a 2010 study, LMNB1 was found to be significantly upregulated in HCC tumours and present in patients' plasma. Also, an increase in the circulating LMNB1 marker in plasma could detect early stage HCC patients with 76% sensitivity and 82% specificity(104).

Therefore, LMNB1 mRNA measurement was proposed in patients with CLD who have normal serum α-F, particularly in known cirrhotic patients who deteriorate rapidly with no apparent cause(105).

LMNB1 has the potential to be both an effective therapeutic target and a reliable prognostic biomarker for HCC(106). LMNB1 was found to promote HCC progression by regulating the phosphatidylinositol 3-kinase (PI3K) and mitogen-activated protein kinase (MAPK) pathways.

1.1.29 IGF

There is over-expression of Insulin-like growth factor II (IGF-II) in HCC tissue. Through activation of IGF1 receptor signalling, IGF2 overexpression accelerates the formation of liver tumours in mice with hepatic expression of MYC and AKT1(107).

Insulin-like growth factor II (IGF-II) levels in serum can be used as a serological marker in the early detection of HCC and to differentiate HCC from cirrhosis(108,109). Furthermore, IGF-2R and IGFBP-2 levels in HCC tissues were higher than in adjacent tissues(110).

A study that looked into the diagnostic use of IGF-II in small HCC discovered that both IGF-II and α-FP levels in HCC were higher than in controls ($P = 0.0001$). The sensitivity, specificity, and diagnostic accuracy values for IGF-II were 63%, 90%, and 70%, respectively(111).

It has also been demonstrated that there is a significant relationship between IGF-1 expression and liver cirrhosis and survival after resection in patients with HCC, regardless of the underlying liver disease(112).

1.1.30 CCT3

Chaperonin-containing TCP-1 3 (CCT3) is required for HCC cell proliferation(113). According to a microarray and RNA-sequencing-based study with 4272 cases, CCT3 may play a role in HCC tumorigenesis and progression, as well as have prognostic value in HCC(114).

CCT3 overexpression because of hypomethylation in cancerous cell nuclei is linked to HCC progression and it may be a target that influences STAT3 activation in HCC(115,116).

CCT3 levels in HCC patients' plasma were significantly higher than in cirrhotic patients and healthy controls ($P < 0.01$)(115). When compared to α-FP, CCT3 protein had a higher sensitivity in the diagnosis of HCC (87.3% vs 69.8%). Furthermore, CCT3 could be useful to detect α-FP-negative HCC patients with sensitivity and specificity of 92.1% and 81.6%, respectively. CCT3 sensitivity was 76.6% for the detection of small HCCs(14).

1.1.31 ATIII

Serum antithrombin III (ATIII) was discovered to have the potential to be useful biomarkers for differentiating α-FP-negative HCC from cirrhosis(29, 31).

ATIII may also be useful in predicting the outcomes of HCC patients after curative hepatectomy(117,118).

1.1.32 ANGPTL2

Angiopoietin-related protein 2 (ANGPTL2) is involved in metabolism, vascular biology, inflammation, and tumour metastasis, but little is known about its role in human HCC metastasis. ANGPTL2 expression was higher in HCC tissues compared to noncancerous liver tissues in HCC patients. Overexpression of ANGPTL2 increased HCC cell migration and invasion in vitro and promoted intrahepatic and distal pulmonary metastasis in vivo(119). Besides, ANGPTL2 has been proposed as a promising biomarker for the diagnosis of CHB-related HCC. A significantly higher serum ANGPTL2 level was detected in HCC than

in healthy controls ($P<0.001$) in a 2019 study enrolling 361 participants in the discovery cohort. The ROC results showed that ANGPTL2 had a significantly higher AUC for predicting HCC than α-FP. Serum ANGPTL2 levels in the validation cohort gradually increased with the progression of chronic hepatitis B virus infection, peaking in HCC. Moreover, combining ANGPTL2 and α-FP may improve diagnostic accuracy for HCC when compared to either ANGPTL2 or α-FP alone(120).

Notably, the expression of some ANGPTL gene family members (except ANGPTL2) has been found to be significantly correlated with HCC prognosis, suggesting that ANGPTL gene family members may be promising molecular markers for HCC treatment and prognosis(121).

1.1.33 Hsp90-α

The findings of a 2010 study demonstrated the ability of the hepatitis B Virus X protein (HBx) to promote tumour cell invasion via a mechanism involving the up-regulation of Heat shock protein 90 (Hsp90-α), providing new insights into the mechanism of action of HBx and its role in tumour metastasis and recurrence of HCC(122).

In another 2020 study of 659 HCC patients, plasma Hsp90-α levels were significantly higher in HCC patients than in controls ($P<0.05$). ROC curve analysis revealed that the combination of Hsp90-α and α-FP (AUC 0.94) significantly improved diagnostic efficiency for HCC patients when compared to α-FP (AUC 0.92) or Hsp90-α (AUC 0.836). As a result, the findings suggest that plasma Hsp90-α levels can be used to make an initial diagnosis of HCC in both rural and urban settings(123).

1.1.34 GDF15

Growth Differentiation Factor-15 (GDF15) is a divergent TGF-beta family member that is expressed after liver injury and carcinogen exposure. GDF-15 expression has been linked to gastrointestinal cancer stage, size, and metastasis, as well as tumour growth inhibition and increased tumour invasiveness (124).

GDF-15 is an effective serum marker for the detection of HBV-related HCC (125). In patients with liver cirrhosis or HCC, GDF15 had a sensitivity of 63.1% and a specificity of 86.6% when compared to HBV or HCV carriers at the optimal cut-off point of 2.46 ng/mL(126).

More recently, it was discovered that Serum GDF15 can predict the occurrence of de novo HCC(127).

Serum GDF15 measurement as a biomarker for HCC and cirrhosis has been recommended for further research and clinical application.

1.1.35 TM4SF1 and TM4SF5

Transmembrane 4 L6 family member 1 (TM4SF1) has been reported to be upregulated in a variety cancer types as a tumor associated antigen. TM4SF1 was found to be associated with human poorly differentiated HCC in deep sequencing and comprehensive expression analysis. However, the functions and mechanisms of TM4SF1 in the promotion of HCC are still unknown(128,129).

It was discovered that TM4SF1 is a direct target of that may act as a tumor suppressor in HCC proliferation and invasion. In HCC samples, there was an inverse relationship between miR-520f and TM4SF1 mRNA levels. The restoration of TM4SF1 partially abolished miR-520f-mediated cell proliferation and invasion inhibition in HCC cells by regulating the P13K/AKT and p38 MAPK signaling pathways(129).

Also, a recent study found that TM4SF1 is an interacting partner of DVL2 and that it positively regulates Wnt/beta-catenin signaling by strengthening the DVL2-Axin interaction. The expression of TM4SF1 was increased in HCC and was induced by Kras signaling. Overexpression of TM4SF1 promoted HCC cell growth and motility while also upregulating target genes such as axin2 and cyclin D1, revealing TM4SF1's oncogenic functions in HCC progression(128).

TM4SF5 has been implicated in the uncontrolled growth of human HCC cells via epithelial-mesenchymal transition in some studies(130).

Some researchers have recently discovered that TM4SF5-mediated STAT3 activity for extracellular matrix modulation is involved in the progression of liver disease to HCC, and that TM4SF5 appears to suppress NK cells during the progression of liver carcinogenesis(131). Other authors proposed that lysosomal TM4SF5 senses and facilitates arginine efflux for mTORC1/S6K1 activation, and that arginine-auxotrophs in HCC could be targeted by blocking arginine sensing with anti-TM4SF5 reagents (132). These findings could provide potential therapeutic evidence of both TM4SF1 and TM4SF5 for HCC patients.

1.2 Immunological Biomarkers

1.1.1 Pentraxin 3

Pentraxin 3 is a protein produced by multiple cell types in response to inflammatory signals, including macrophages, monocytes, fibroblasts, and endothelial cells; as such, pentraxins act as acute-phase proteins and as an innate immune system component. Pentraxin 3 may also play a role in cancer development, though the underlying mechanisms are unknown.

Pentraxin 3 levels have been found to be elevated in patients suffering from acute liver injury, NASH, and HCV, among other conditions(133).

In a recent Chinese study, serum pentraxin 3 was proposed as a candidate biomarker of HBV-induced HCC(134). A comparison of serum pentraxin 3 levels in 107 HCC patients to 159 chronic HBV and 99 cirrhotic patients revealed that pentraxin 3 was highly discriminative of α-FP-negative and early-stage HCC, and its diagnostic performance was superior to α-FP ($P< 0.001$). Pentraxin 3 was discovered to be an independent risk factor for HCC (OR 1.62) and to be capable of distinguishing HCC in CHB (cut-off 9.23 ng/mL, AUROC 93%), including α-FP negative (cut-off 8.98 ng/mL, AUROC 95%) and early-stage HCC (cut-off 9.36 ng/mL, AUROC 92%)(134).

In summary, pentraxin-3 measurement in CHB could detect HCC, including α-FP-negative and early-stage HCC.

1.1.2 TEMs

Angiogenesis is a critical step in the development and progression of HCC. Myeloid lineage cells, such as macrophages and monocytes, have been shown to regulate angiogenesis in mouse tumour models. TIE2, an angiopoietin receptor, specifically transmits pro-angiogenic signals and identifies a pro-angiogenic monocyte/macrophage subset(135). As a result of the increased frequency of TIE2-expressing monocytes/macrophages (TEMs) in HCC patients, some authors have proposed that TEM frequency can be used as a diagnostic marker for HCC, potentially reflecting liver angiogenesis(135).

ROC curves revealed that the optimal diagnostic cut-off value for TEMs was 4.95% in a 2017 Chinese study with 190 participants. The proportion of TEMs in peripheral CD14+CD16+ monocytes in the HCC group was significantly higher than in the CHB, HBV-related liver cirrhosis, and healthy control groups ($P < 0.05$). In the α-FP-negative HCC group, the percentage of TEMs was also significantly higher than in the other groups ($P < 0.05$)(136). The AUROC for TEMs in distinguishing α-FP-negative HCC from HBV-related liver cirrhosis was 70%, with a sensitivity of 80.0% and a specificity of 65.52%. In univariate and multivariate analyses, only TEMs were found to be a significant predictor of α-FP-negative HCC ($P = 0.016$, $P = 0.023$, respectively)(136).

More evidence is needed, however, to confirm TEM accuracy in the early detection of HCC, particularly in α-FP-negative cases and other CLD aetiologies.

1.1.3 VersicanV1

Versican, a key extracellular matrix regulator of immunity and inflammation, has been linked to carcinogenesis in a variety of cancers(137).

However, the precise role of VersicanV1, the most common versican isoform in the liver, in HCC is unknown.

HCC patients who had positive VersicanV1 coexpression had a worse prognosis(138).

VersicanV1, which is regulated by direct interaction with Linc01225, was found to be significantly upregulated in HCC tissues and correlates with poor prognosis via the EGFR-PI3K-AKT pathway activation in a 2020 study(139).

1.1.4 MIG

Monokine Induced by Gamma Interferon-6 (MIG-6) ablation has been shown to cause tumour formation in a variety of tissues.

Mig-6 expression in HCC is low, indicating a poor prognosis. MIG-6 may influence cell proliferation and the cell cycle via the P-ERK/Cyclin D1 signalling pathway(140).

1.1.5 DHCR24 Antibody

Serum 3b-hydroxysterol 124-reductase antibody (DHCR24) is an autoimmune protein that is significantly upregulated in HCV patients and can be used as a diagnostic biomarker for HCC associated with CHC(141), with a higher AUROC than α-FP and DCP in distinguishing HCV-mediated chronic hepatitis from HCV-mediated HCC patients. Hence, serum DHCR24 antibody could be a biomarker for the diagnosis of HCV-related HCC with negative α-FP.

1.1.6 IL-22

Interleukin-22 (IL-22) exhibits both protective and pathological properties in liver diseases(142). In CHB patients, IL-22 promotes the progression of HCC. High levels of tumour-infiltrating IL-22+ cells and serum IL-22 are thought to be poor prognostic indicators for HCC.

1.2 Genetic Biomarkers

1.2.10 MCM6

Minichromosome maintenance complex component 6 (MCM6) is an important DNA replication regulator that is essential for cell cycle maintenance. MCM6 expression is increased in many cancer cells(143).

MCM6, has been identified as a S/G2 cell cycle progression driver as well as a potential diagnostic and prognostic marker in HCC. MCM6 outperformed MCM2 and MCM7 in HCC diagnostic performance (AUROC 89.6% vs. 67.5 and 77.1%, $P < 0.01$)(144-146).

MCM6 promoted epithelial-mesenchymal transition and activated MEK/ERK signaling, according to mechanistic analyses. More importantly, serum MCM6 levels in HCC patients were significantly higher than in cirrhosis and healthy controls ($P < 0.0001$), and allowed for high accuracy in distinguishing early recurrence (AUROC, 77%)(147).

1.2.11 AREG

Amphiregulin (AREG) is an EGFR ligand that plays a relevant role in cell proliferation, survival, and migration(148). AREG expression is indeed very low in normal liver, but it increases dramatically after liver injury, resulting in a critical pro-regenerative function. Nevertheless, an aggravated tissue repair reaction may have adverse implications because AREG induces an autocrine loop that keeps HCC cells alive(149).

Oestrogens may also increase AREG expression in human HCC, and locally elevated aromatase activity may increase malignant cell proliferation via AREG signalling(150,151). The AREG/EGFR system was discovered to be a key mediator of FGF19 responses in HCC cells via β-catenin signalling(152).

In a 2019 Egyptian study that included only 55 HCC patients, 20 cirrhotic patients, and 15 healthy subjects, AREG had a sensitivity of 74.5% but low specificity of 47.1% at a threshold of 8.74 pg/ml(149).

Furthermore, in HCC patients, AREG was associated with portal vein thrombosis and tumour metastasis(149). Therefore, the lack of clinical data on AREG accuracy prevents us from commenting on its HCC detection potential.

2.0 Combination of less validated HCC biomarkers

Altered glycosylation of serum glycoproteins can act as potential biomarkers for detection of HCC when used alone or in combination with other HCC markers. In a 1999 Japanese study, the combination of fucosylated α-FP measurements with alpha-1-antitrypsin or transferrin was useful for the diagnosis of HCC(108). The combination of fucosylated kininogen, α-FP, and GP73 produced the best results, with an optimal sensitivity of 95%, specificity of 70%, and AUROC of 94%(109).

In a 2017 Chinese study, the combination of TEMs, DKK1, and α-FP measurements increased the AUROC for HCC diagnosis (83%; 95% CI 77%-89%). As a result, the authors concluded that TEMs and DKK1 could be complementary biomarkers for α-FP in the diagnosis of HCC, and that TEMs, rather than DKK1, could serve as a complementary biomarker for α-FP in the differential diagnosis of α-FP-negative HCC versus HBV-related liver cirrhosis patients(136).

The combination of α-FP and AGP could aid in the diagnosis of HCC. When α-FP levels were less than 500 ng/ml, AGP was useful for distinguishing HCC cases from liver cirrhosis patients. The AUROC of AGP and the combination of AGP and α-FP were 83.4% ($P < 0.0005$) and 88% ($P < 0.0005$), respectively, higher than α-FP alone (0.54, $P = 0.60$)(150). Besides, a simple score based on AGP, CRP, α-FP, and albumin could improve HCC diagnosis accuracy. Their combination had a higher AUROC of 92% and sensitivity of 85% than α-FP alone. With an OR of 50.6 for HCC, the overall score predicted HCC(151). Furthermore, a combination of S2-bound AGP, α-FP, and AGP concentration demonstrated AUROC of 87% and 95%, respectively(152). The panel had the greatest benefit in detecting NASH-related HCCs when combined with INR and α-FP, with a significantly improved AUROC of 88% for all NASH HCCs and 0.82 for early NASH HCCs when compared to α-FP alone (0.76 and 0.64, respectively)(98).

It has been suggested that HCCR-1 may be used in addition to α-FP to detect HCC. Sensitivities for HCC increased to 77.2% when α-FP was combined with HCCR-1. Furthermore, using α-FP and HCCR-1 together improved the diagnostic rate to 70.8% in small HCC (⩾ 2 cm) and 81.6% in large HCC (< 2 cm), respectively(99). Therefore, HCCR-1 may be a useful biomarker for HCC, and the diagnostic rate may be significantly improved when HCCR-1 and α-FP are used together. In another prospective cohort study of 1,338 HCC patients, the combined use of α-FP and HCCR-1 increased the positive rate for HCC to 74.1%. The combined analysis of α-FP and HCCR-1 increased the diagnostic rate for small HCC (< 2 cm) to 56.9%, up from 40.1% and 23.4% in the single analyses of HCCR-1 and α-FP, respectively(153).

According to a 2019 retrospective study HCC diagnosis could be improved by using both serum AKR1B10 and α-FP predictors(154). Measuring serum AKR1B10 and α-FP at the same time increased sensitivity and negative predictive value for HCC diagnosis(155).

When compared to either test alone, the combination of PON1 and α-FP improved diagnostic accuracy for vascular invasion (AUROC 78%, sensitivity 75.96%, specificity 77.44%; PON1 plus α-FP vs. PON1 alone, $P = 0.0004$; PON1 plus α-FP vs. α-FP alone, $P<0.0001$)(6). A total of 1,396 participants a panel of five proteins (OPN, GDF15, NSE, TRAP5 and OPG) demonstrated high

diagnostic accuracy when distinguishing early-stage HCC from the at-risk group, with AUROC of 89%, 91%, and 85% for the training, validation, and cohort 2 data sets, respectively. P5's sensitivity for diagnosing preclinical HCC increased with time in the prediction set (156). Furthermore, an 11-SRG signature (CDX2, PON1, ADH4, RBP2, LCAT, GAL, LPA, CYP19A1, GAST, SST, and UGT1A8) has been identified as a novel prognostic marker for survival prognosis in patients with HCC (157). Additionally, a signature of six DE-ERGs (PPARGC1A, SQSTM1, SGK1, PON1, CDK1, and G6PD) may be a useful tool for prognosis prediction and personal management of HCC patients(158).

When PON1 and ATIII were combined in a 2021 retrospective study with a discovery set of 36 patients and a validation set of 90 patients, the AUROC was 84.8% (sensitivity: 80.0%; specificity: 73.3%), which was significantly better than a single biomarker. PON1 and ATIII may be useful biomarkers for distinguishing α-FP-negative HCC from cirrhosis, according to these findings(3).

CCT3 and IQGAP3 are complementary biomarkers for HCC screening and diagnosis, particularly in α-FP-negative and small HCC patients. α-FP + CCT3 + IQGAP3 had significantly better discriminative ability than α-FP alone (0.95 vs. 0.81; $P < 0.01$)(14).

Differentially expressed genes associated with RPS16, RPS7, CCT3, HNRNPA2B1, EIF4G1, PSMC4, NHP2, EGR1, FDPS, and MCM4 genes and pathways may have diagnostic value as potential biomarkers involved in the pathogenesis of HCC, relating to both obesity and HCC occurrence/recurrence(159). Also, the prognostic gene signature (IQGAP3, BIRC5, PTTG1, STC2, CDKN3, PBK, EXO1, NEIL3, and HOXD9) developed on nine genes can be used as a combined biomarker for the independent prediction of overall survival in HCC patients(160).

Finally, a 2021 study of 100 HCC, cirrhosis, and control patients found that HCC patients had significantly higher VEGF/platelet levels than non-HCC groups ($P = 0.001$). Serum VEGF/platelet levels correlated significantly with HCC tumour size, stage, vascular invasion, and Child-Pugh classification. Furthermore, the sensitivity and specificity of the VEGF/platelet ratio were 77.5% and 80%, respectively, which were higher than the accuracy provided by α-FP. Serum VEGF/platelet, alone or in combination with α-FP, were reliable biomarkers for early and accurate HCC diagnosis in HCV patients(161).

3.0 References

1. Zhang Y, Ying X, Zhao Q, Ma J, Zhang D, He C, et al. Identification of Protein Expression Changes in Hepatocellular Carcinoma through iTRAQ. Dis Markers. 2020;2020:2632716.
2. Sun C, Chen P, Chen Q, Sun L, Kang X, Qin X, et al. Serum paraoxonase 1 heteroplasmon, a fucosylated, and sialylated glycoprotein in distinguishing early hepatocellular carcinoma from liver cirrhosis patients. Acta Biochim Biophys Sin (Shanghai). 2012;44(9):765-73.
3. Cao X, Cao Z, Ou C, Zhang L, Chen Y, Li Y, et al. Combination of serum paraoxonase/arylesterase 1 and antithrombin-III is a promising non-invasion biomarker for discrimination of AFP-negative HCC versus liver cirrhosis patients. Clin Res Hepatol Gastroenterol. 2021;45(5):101583.
4. Cao X, Cao Z, Shao Y, Liu C, Yan G, Meng X, et al. Analysis of Serum Paraoxonase 1 Using Mass Spectrometry and Lectin Immunoassay in Patients With Alpha-Fetoprotein Negative Hepatocellular Carcinoma. Front Oncol. 2021;11:651421.
5. Shu H, Li W, Shang S, Qin X, Zhang S, Liu Y. Diagnosis of AFP-negative early-stage hepatocellular carcinoma using Fuc-PON1. Discov Med. 2017;23(126):163-8.
6. Ding GY, Zhu XD, Ji Y, Shi GM, Shen YH, Zhou J, et al. Serum PON1 as a biomarker for the estimation of microvascular invasion in hepatocellular carcinoma. Ann Transl Med. 2020;8(5):204.
7. Chen M, Zheng T, Han S, Zhang L, Bai Y, Fang X, et al. A preliminary study of plasma cyclase-associated protein 2 as a novel biomarker for early stage and alpha-fetoprotein negative hepatocellular carcinoma patients. Clin Res Hepatol Gastroenterol. 2015;39(2):215-21.
8. Li L, Fu LQ, Wang HJ, Wang YY. CAP2 is a Valuable Biomarker for Diagnosis and Prognostic in Patients with Gastric Cancer. Pathol Oncol Res. 2020;26(1):273-9.
9. Sakamoto M, Mori T, Masugi Y, Effendi K, Rie I, Du W. Candidate molecular markers for histological diagnosis of early hepatocellular carcinoma. Intervirology. 2008;51 Suppl 1:42-5.
10. Fu J, Li M, Wu DC, Liu LL, Chen SL, Yun JP. Increased Expression of CAP2 Indicates Poor Prognosis in Hepatocellular Carcinoma. Transl Oncol. 2015;8(5):400-6.
11. Dai Q, Ain Q, Rooney M, Song F, Zipprich A. Role of IQ Motif-Containing GTPase-Activating Proteins in Hepatocellular Carcinoma. Front Oncol. 2022;12:920652.
12. Shi Y, Qin N, Zhou Q, Chen Y, Huang S, Chen B, et al. Role of IQGAP3 in metastasis and epithelial-mesenchymal transition in human hepatocellular carcinoma. J Transl Med. 2017;15(1):176.
13. Lin M, Liu Y, Ding X, Ke Q, Shi J, Ma Z, et al. E2F1 transactivates IQGAP3 and promotes proliferation of hepatocellular carcinoma cells through IQGAP3-mediated PKC-alpha activation. Am J Cancer Res. 2019;9(2):285-99.
14. Qian EN, Han SY, Ding SZ, Lv X. Expression and diagnostic value of CCT3 and IQGAP3 in hepatocellular carcinoma. Cancer Cell Int. 2016;16:55.
15. Wong KF, Luk JM. Discovery of lamin B1 and vimentin as circulating biomarkers for early hepatocellular carcinoma. Methods Mol Biol. 2012;909:295-310.
16. You H, Yuan D, Bi Y, Zhang N, Li Q, Tu T, et al. Hepatitis B virus X protein promotes vimentin expression via LIM and SH3 domain protein 1 to facilitate epithelial-mesenchymal transition and hepatocarcinogenesis. Cell Commun Signal. 2021;19(1):33.
17. Zhai X, Zhu H, Wang W, Zhang S, Zhang Y, Mao G. Abnormal expression of EMT-related proteins, S100A4, vimentin and E-cadherin, is correlated with clinicopathological features and prognosis in HCC. Med Oncol. 2014;31(6):970.
18. Idriss NK, Fakhry M, Imam HM, Abd-Elmoez FA, Abdelewahab H, Abdel-Wahid L, et al. Analysis of Lamin B1, Vimentin and Anti-Ku86 as Prospective Biomarkers of Hepatocellular Carcinoma in Patients with Hepatitis C Virus Infection. Cell Physiol Biochem. 2019;52(3):595-605.
19. Spadaro A, Tortorella V, Morace C, Fortiguerra A, Composto P, Bonfiglio C, et al. High circulating D-dimers are associated with ascites and hepatocellular carcinoma in liver cirrhosis. World J Gastroenterol. 2008;14(10):1549-52.
20. Liu Z, Guo H, Gao F, Shan Q, Li J, Xie H, et al. Fibrinogen and D-dimer levels elevate in advanced hepatocellular carcinoma: High pretreatment fibrinogen levels predict poor outcomes. Hepatol Res. 2017;47(11):1108-17.

21. Malaguarnera M, Latteri S, Bertino G, Madeddu R, Catania VE, Curro G, et al. D-dimer plasmatic levels as a marker for diagnosis and prognosis of hepatocellular carcinoma patients with portal vein thrombosis. Clin Exp Gastroenterol. 2018;11:373-80.
22. Fang P, Du L, Cai D. Evaluation of plasma D-dimer for the diagnosis in Chinese patients with hepatocellular carcinoma: A meta-analysis. Medicine (Baltimore). 2020;99(12):e19461.
23. Peng F, Yuan H, Zhou YF, Wu SX, Long ZY, Peng YM. Diagnostic Value of Combined Detection via Protein Induced by Vitamin K Absence or Antagonist II, Alpha-Fetoprotein, and D-Dimer in Hepatitis B Virus-Related Hepatocellular Carcinoma. Int J Gen Med. 2022;15:5763-73.
24. Naitoh A, Aoyagi Y, Asakura H. Highly enhanced fucosylation of serum glycoproteins in patients with hepatocellular carcinoma. J Gastroenterol Hepatol. 1999;14(5):436-45.
25. Comunale MA, Wang M, Hafner J, Krakover J, Rodemich L, Kopenhaver B, et al. Identification and development of fucosylated glycoproteins as biomarkers of primary hepatocellular carcinoma. J Proteome Res. 2009;8(2):595-602.
26. Wang M, Long RE, Comunale MA, Junaidi O, Marrero J, Di Bisceglie AM, et al. Novel fucosylated biomarkers for the early detection of hepatocellular carcinoma. Cancer Epidemiol Biomarkers Prev. 2009;18(6):1914-21.
27. Shang S, Qin X, Li W, Zhang S, Liu Y. ELISA index of serum fucosylated haptoglobin for diagnosis of HCC using the normal and reverse AAL ELISA. Discov Med. 2016;21(113):15-23.
28. Liao J, Zhang R, Qian H, Cao L, Zhang Y, Xu W, et al. Serum profiling based on fucosylated glycoproteins for differentiating between chronic hepatitis B and hepatocellular carcinoma. Biochem Biophys Res Commun. 2012;420(2):308-14.
29. Zhu J, Lin Z, Wu J, Yin H, Dai J, Feng Z, et al. Analysis of serum haptoglobin fucosylation in hepatocellular carcinoma and liver cirrhosis of different etiologies. J Proteome Res. 2014;13(6):2986-97.
30. Kobayashi S, Nouso K, Kinugasa H, Takeuchi Y, Tomoda T, Miyahara K, et al. Clinical utility of serum fucosylated hemopexin in Japanese patients with hepatocellular carcinoma. Hepatol Res. 2012;42(12):1187-95.
31. Morota K, Nakagawa M, Sekiya R, Hemken PM, Sokoll LJ, Elliott D, et al. A comparative evaluation of Golgi protein-73, fucosylated hemopexin, alpha-fetoprotein, and PIVKA-II in the serum of patients with chronic hepatitis, cirrhosis, and hepatocellular carcinoma. Clin Chem Lab Med. 2011;49(4):711-8.
32. Asazawa H, Kamada Y, Takeda Y, Takamatsu S, Shinzaki S, Kim Y, et al. Serum fucosylated haptoglobin in chronic liver diseases as a potential biomarker of hepatocellular carcinoma development. Clin Chem Lab Med. 2015;53(1):95-102.
33. Tanabe K, Kitagawa K, Kojima N, Iijima S. Multifucosylated Alpha-1-acid Glycoprotein as a Novel Marker for Hepatocellular Carcinoma. J Proteome Res. 2016;15(9):2935-44.
34. Yao W, Wang K, Jiang Y, Huang Z, Huang Y, Yan H, et al. Serum profile of low molecular weight fucosylated glycoproteins for early diagnosis of hepatocellular carcinoma. Oncol Lett. 2020;20(2):1597-606.
35. Sandhu DS, Baichoo E, Roberts LR. Fibroblast growth factor signaling in liver carcinogenesis. Hepatology. 2014;59(3):1166-73.
36. Mise M, Arii S, Higashituji H, Furutani M, Niwano M, Harada T, et al. Clinical significance of vascular endothelial growth factor and basic fibroblast growth factor gene expression in liver tumor. Hepatology. 1996;23(3):455-64.
37. Gauglhofer C, Sagmeister S, Schrottmaier W, Fischer C, Rodgarkia-Dara C, Mohr T, et al. Up-regulation of the fibroblast growth factor 8 subfamily in human hepatocellular carcinoma for cell survival and neoangiogenesis. Hepatology. 2011;53(3):854-64.
38. Wang H, Yang J, Zhang K, Liu J, Li Y, Su W, et al. Advances of Fibroblast Growth Factor/Receptor Signaling Pathway in Hepatocellular Carcinoma and its Pharmacotherapeutic Targets. Front Pharmacol. 2021;12:650388.
39. Uematsu S, Higashi T, Nouso K, Kariyama K, Nakamura S, Suzuki M, et al. Altered expression of vascular endothelial growth factor, fibroblast growth factor-2 and endostatin in patients with hepatocellular carcinoma. J Gastroenterol Hepatol. 2005;20(4):583-8.

40. Miura S, Mitsuhashi N, Shimizu H, Kimura F, Yoshidome H, Otsuka M, et al. Fibroblast growth factor 19 expression correlates with tumor progression and poorer prognosis of hepatocellular carcinoma. BMC Cancer. 2012;12:56.
41. Raja A, Park I, Haq F, Ahn SM. FGF19-FGFR4 Signaling in Hepatocellular Carcinoma. Cells. 2019;8(6).
42. Chen Z, Jiang L, Liang L, Koral K, Zhang Q, Zhao L, et al. The Role of Fibroblast Growth Factor 19 in Hepatocellular Carcinoma. Am J Pathol. 2021;191(7):1180-92.
43. Mohamed GA, Nashaat EH, Fawzy HM, ElGhandour AM. Assessment of fibroblast growth factor 19 as a non-invasive serum marker for hepatocellular carcinoma. World J Hepatol. 2022;14(3):623-33.
44. Li Y, Yu S, Li L, Chen J, Quan M, Li Q, et al. KLF4-mediated upregulation of CD9 and CD81 suppresses hepatocellular carcinoma development via JNK signaling. Cell Death Dis. 2020;11(4):299.
45. Sun H, Peng Z, Tang H, Xie D, Jia Z, Zhong L, et al. Loss of KLF4 and consequential downregulation of Smad7 exacerbate oncogenic TGF-beta signaling in and promote progression of hepatocellular carcinoma. Oncogene. 2017;36(21):2957-68.
46. Chen J, Zhang L, Ma S, Lu G, Wang D. The aberrant expressions of MACC1, ZEB1, and KLF4 in hepatocellular carcinoma and their clinical significance. Int J Clin Exp Pathol. 2019;12(9):3653-61.
47. Nomura F, Sogawa K, Noda K, Seimiya M, Matsushita K, Miura T, et al. Serum anti-Ku86 is a potential biomarker for early detection of hepatitis C virus-related hepatocellular carcinoma. Biochem Biophys Res Commun. 2012;421(4):837-43.
48. Idriss NK, Fakhry M, Imam HM, Abd-Elmoez FA, Abdelewahab H, Abdel-Wahid L, et al. Analysis of Lamin B1, Vimentin and Anti-Ku86 as Prospective Biomarkers of Hepatocellular Carcinoma in Patients with Hepatitis C Virus Infection. Cell Physiol Biochem. 2019;52(3):595-605.
49. Yi CH, Weng HL, Zhou FG, Fang M, Ji J, Cheng C, et al. Elevated core-fucosylated IgG is a new marker for hepatitis B virus-related hepatocellular carcinoma. Oncoimmunology. 2015;4(12):e1011503.
50. Inui Y, Higashiyama S, Kawata S, Tamura S, Miyagawa J, Taniguchi N, et al. Expression of heparin-binding epidermal growth factor in human hepatocellular carcinoma. Gastroenterology. 1994;107(6):1799-804.
51. Huang P, Xu X, Wang L, Zhu B, Wang X, Xia J. The role of EGF-EGFR signalling pathway in hepatocellular carcinoma inflammatory microenvironment. J Cell Mol Med. 2014;18(2):218-30.
52. Liu Z, Chen D, Ning F, Du J, Wang H. EGF is highly expressed in hepatocellular carcinoma (HCC) and promotes motility of HCC cells via fibronectin. J Cell Biochem. 2018;119(5):4170-83.
53. Wang J, Zhong Y, Meng G. EGF rs4444903 polymorphism is associated with risk of HCV-related cirrhosis and HBV/HCV-related hepatocellular carcinoma. Int J Clin Oncol. 2021;26(11):2053-64.
54. Wang Q, Xu L, Wu Q, Zhang M, Zhang J. Association between the risk of hepatitis virus-related hepatocellular carcinoma and EGF polymorphism: A PRISMA-compliant updated meta-analysis. Medicine (Baltimore). 2022;101(42):e31280.
55. Abbas E, Shaker O, Abd El Aziz G, Ramadan H, Esmat G. Epidermal growth factor gene polymorphism 61A/G in patients with chronic liver disease for early detection of hepatocellular carcinoma: a pilot study. Eur J Gastroenterol Hepatol. 2012;24(4):458-63.
56. Yamagamim H, Moriyama M, Matsumura H, Aoki H, Shimizu T, Saito T, et al. Serum concentrations of human hepatocyte growth factor is a useful indicator for predicting the occurrence of hepatocellular carcinomas in C-viral chronic liver diseases. Cancer. 2002;95(4):824-34.
57. Youness ER, El Nemr M, Oraby FS, Ahmed NM, Moghni MA, Aly HF, et al. Evaluation of Apoptotic Marker Bcl2, CD4+, Human Hepatocyte Growth Factor and Metalloproteinase-9 as Tumor Markers for Patients with Hepatocellular Carcinoma. Indian J Clin Biochem. 2014;29(3):351-6.
58. Huang X, Gan G, Wang X, Xu T, Xie W. The HGF-MET axis coordinates liver cancer metabolism and autophagy for chemotherapeutic resistance. Autophagy. 2019;15(7):1258-79.
59. Pocino K, Napodano C, Marino M, Di Santo R, Miele L, De Matthaeis N, et al. A Comparative Study of Serum Angiogenic Biomarkers in Cirrhosis and Hepatocellular Carcinoma. Cancers (Basel). 2021;14(1).
60. Karabulut S, Tas F, Akyuz F, Ormeci AC, Serilmez M, Soydinc HO, et al. Clinical significance of serum hepatocyte growth factor (HGF) levels in hepatocellular carcinoma. Tumour Biol. 2014;35(3):2327-33.
61. Yamaguchi R, Yano H, Iemura A, Ogasawara S, Haramaki M, Kojiro M. Expression of vascular endothelial growth factor in human hepatocellular carcinoma. Hepatology. 1998;28(1):68-77.

62. Kong SY, Park JW, Lee JA, Park JE, Park KW, Hong EK, et al. Association between vascular endothelial growth factor gene polymorphisms and survival in hepatocellular carcinoma patients. Hepatology. 2007;46(2):446-55.
63. Schoenleber SJ, Kurtz DM, Talwalkar JA, Roberts LR, Gores GJ. Prognostic role of vascular endothelial growth factor in hepatocellular carcinoma: systematic review and meta-analysis. Br J Cancer. 2009;100(9):1385-92.
64. El-Sherif WT, Makhlouf NA, El-Gendi SS, Hassan HI, Herdan OM. Evaluation of transforming growth factor alpha and vascular endothelial growth factor in diagnosis of hepatocellular carcinoma. Egypt J Immunol. 2012;19(2):53-65.
65. Mukozu T, Nagai H, Matsui D, Kanekawa T, Sumino Y. Serum VEGF as a tumor marker in patients with HCV-related liver cirrhosis and hepatocellular carcinoma. Anticancer Res. 2013;33(3):1013-21.
66. Lacin S, Yalcin S. The Prognostic Value of Circulating VEGF-A Level in Patients With Hepatocellular Cancer. Technol Cancer Res Treat. 2020;19:1533033820971677.
67. Yamaguchi R, Yano H, Nakashima O, Akiba J, Nishida N, Kurogi M, et al. Expression of vascular endothelial growth factor-C in human hepatocellular carcinoma. J Gastroenterol Hepatol. 2006;21(1 Pt 1):152-60.
68. Alzamzamy A, Elsayed H, Abd Elraouf M, Eltoukhy H, Megahed T, Aboubakr A. Serum vascular endothelial growth factor as a tumor marker for hepatocellular carcinoma in hepatitis C virus-related cirrhotic patients. World J Gastrointest Oncol. 2021;13(6):600-11.
69. Fernandes-Ferreira R, Tenani GD, Pinhel MAS, Abrantes AMC, Botelho M, Silva R, et al. Genes Expression and Serum Biomarkers for Diagnosis of Hepatocellular Carcinoma, Cirrhosis and Hepatitis C. Arq Gastroenterol. 2022;59(3):394-401.
70. Sugimachi K, Tanaka S, Taguchi K, Aishima S, Shimada M, Tsuneyoshi M. Angiopoietin switching regulates angiogenesis and progression of human hepatocellular carcinoma. J Clin Pathol. 2003;56(11):854-60.
71. Abdel Ghafar MT, Elkhouly RA, Elnaggar MH, Mabrouk MM, Darwish SA, Younis RL, et al. Utility of serum neuropilin-1 and angiopoietin-2 as markers of hepatocellular carcinoma. J Investig Med. 2021;69(6):1222-9.
72. Mitsuhashi N, Shimizu H, Ohtsuka M, Wakabayashi Y, Ito H, Kimura F, et al. Angiopoietins and Tie-2 expression in angiogenesis and proliferation of human hepatocellular carcinoma. Hepatology. 2003;37(5):1105-13.
73. Choi GH, Jang ES, Kim JW, Jeong SH. Prognostic role of plasma level of angiopoietin-1, angiopoietin-2, and vascular endothelial growth factor in hepatocellular carcinoma. World J Gastroenterol. 2021;27(27):4453-67.
74. Dong ZZ, Yao DF, Yao M, Qiu LW, Zong L, Wu W, et al. Clinical impact of plasma TGF-beta1 and circulating TGF-beta1 mRNA in diagnosis of hepatocellular carcinoma. Hepatobiliary Pancreat Dis Int. 2008;7(3):288-95.
75. Lu WQ, Qiu JL, Huang ZL, Liu HY. Enhanced circulating transforming growth factor beta 1 is causally associated with an increased risk of hepatocellular carcinoma: a mendelian randomization meta-analysis. Oncotarget. 2016;7(51):84695-704.
76. Ibrahim GH, Mahmoud MA, Aly NM. Evaluation of circulating Transforming growth factor-beta1, Glypican-3 and Golgi protein-73 mRNAs expression as predictive markers for hepatocellular carcinoma in Egyptian patients. Mol Biol Rep. 2013;40(12):7069-75.
77. Peng L, Yuan XQ, Zhang CY, Ye F, Zhou HF, Li WL, et al. High TGF-beta1 expression predicts poor disease prognosis in hepatocellular carcinoma patients. Oncotarget. 2017;8(21):34387-97.
78. Devan AR, Pavithran K, Nair B, Murali M, Nath LR. Deciphering the role of transforming growth factor-beta 1 as a diagnostic-prognostic-therapeutic candidate against hepatocellular carcinoma. World J Gastroenterol. 2022;28(36):5250-64.
79. Bhattacharya SD, Garrison J, Guo H, Mi Z, Markovic J, Kim VM, et al. Micro-RNA-181a regulates osteopontin-dependent metastatic function in hepatocellular cancer cell lines. Surgery. 2010;148(2):291-7.
80. Farra R, Grassi G, Tonon F, Abrami M, Grassi M, Pozzato G, et al. The Role of the Transcription Factor E2F1 in Hepatocellular Carcinoma. Curr Drug Deliv. 2017;14(2):272-81.

81. Qiao L, Zhang Q, Sun Z, Liu Q, Wu Z, Hu W, et al. The E2F1/USP11 positive feedback loop promotes hepatocellular carcinoma metastasis and inhibits autophagy by activating ERK/mTOR pathway. Cancer Lett. 2021;514:63-78.
82. Wang SN, Wang LT, Sun DP, Chai CY, Hsi E, Kuo HT, et al. Intestine-specific homeobox (ISX) upregulates E2F1 expression and related oncogenic activities in HCC. Oncotarget. 2016;7(24):36924-39.
83. Gonzalez-Romero F, Mestre D, Aurrekoetxea I, O'Rourke CJ, Andersen JB, Woodhoo A, et al. E2F1 and E2F2-Mediated Repression of CPT2 Establishes a Lipid-Rich Tumor-Promoting Environment. Cancer Res. 2021;81(11):2874-87.
84. Zheng Y, Shi Y, Yu S, Han Y, Kang K, Xu H, et al. GTSE1, CDC20, PCNA, and MCM6 Synergistically Affect Regulations in Cell Cycle and Indicate Poor Prognosis in Liver Cancer. Anal Cell Pathol (Amst). 2019;2019:1038069.
85. Zhang S, Xie C, Li H, Zhang K, Li J, Wang X, et al. Ubiquitin-specific protease 11 serves as a marker of poor prognosis and promotes metastasis in hepatocellular carcinoma. Lab Invest. 2018;98(7):883-94.
86. Yang H, Park D, Ryu J, Park T. USP11 degrades KLF4 via its deubiquitinase activity in liver diseases. J Cell Mol Med. 2021;25(14):6976-87.
87. Zhang C, Xie C, Wang X, Huang Y, Gao S, Lu J, et al. Aberrant USP11 expression regulates NF90 to promote proliferation and metastasis in hepatocellular carcinoma. Am J Cancer Res. 2020;10(5):1416-28.
88. Sun Y, Mi W, Cai J, Ying W, Liu F, Lu H, et al. Quantitative proteomic signature of liver cancer cells: tissue transglutaminase 2 could be a novel protein candidate of human hepatocellular carcinoma. J Proteome Res. 2008;7(9):3847-59.
89. Ma H, Xie L, Zhang L, Yin X, Jiang H, Xie X, et al. Activated hepatic stellate cells promote epithelial-to-mesenchymal transition in hepatocellular carcinoma through transglutaminase 2-induced pseudohypoxia. Commun Biol. 2018;1:168.
90. Yamaguchi H, Kuroda K, Sugitani M, Takayama T, Hasegawa K, Esumi M. Transglutaminase 2 is upregulated in primary hepatocellular carcinoma with early recurrence as determined by proteomic profiles. Int J Oncol. 2017;50(5):1749-59.
91. Ikeda Y, Imai Y, Kumagai H, Nosaka T, Morikawa Y, Hisaoka T, et al. Vasorin, a transforming growth factor beta-binding protein expressed in vascular smooth muscle cells, modulates the arterial response to injury in vivo. Proc Natl Acad Sci U S A. 2004;101(29):10732-7.
92. Liang W, Guo B, Ye J, Liu H, Deng W, Lin C, et al. Vasorin stimulates malignant progression and angiogenesis in glioma. Cancer Sci. 2019;110(8):2558-72.
93. Li S, Li H, Yang X, Wang W, Huang A, Li J, et al. Vasorin is a potential serum biomarker and drug target of hepatocarcinoma screened by subtractive-EMSA-SELEX to clinic patient serum. Oncotarget. 2015;6(12):10045-59.
94. Wang F, Huang J, Zhu Z, Ma X, Cao L, Zhang Y, et al. Transcriptome Analysis of WHV/c-myc Transgenic Mice Implicates Cytochrome P450 Enzyme 17A1 as a Promising Biomarker for Hepatocellular Carcinoma. Cancer Prev Res (Phila). 2016;9(9):739-49.
95. Huang X, Xiang L, Wang B, Hu J, Liu C, Ren A, et al. CMTM6 promotes migration, invasion, and EMT by interacting with and stabilizing vimentin in hepatocellular carcinoma cells. J Transl Med. 2021;19(1):120.
96. Zhu X, Qi G, Li C, Bei C, Tan C, Zhang Y, et al. Expression and Clinical Significance of CMTM6 in Hepatocellular Carcinoma. DNA Cell Biol. 2019;38(2):193-7.
97. Zhang D, Huang J, Luo D, Feng X, Liu Y, Liu Y. Glycosylation change of alpha-1-acid glycoprotein as a serum biomarker for hepatocellular carcinoma and cirrhosis. Biomark Med. 2017;11(5):423-30.
98. Liang J, Zhu J, Wang M, Singal AG, Odewole M, Kagan S, et al. Evaluation of AGP Fucosylation as a Marker for Hepatocellular Carcinoma of Three Different Etiologies. Sci Rep. 2019;9(1):11580.
99. Jirun P, Zhang G, Kim HK, Ha SA, Zhongtian J, Shishi Q, et al. Clinical utility of alpha fetoprotein and HCCR-1, alone or in combination, in patients with chronic hepatitis, liver cirrhosis and hepatocellular carcinoma. Dis Markers. 2011;30(6):307-15.

100. Zhao FT, Jia ZS, Yang Q, Song L, Jiang XJ. S100A14 promotes the growth and metastasis of hepatocellular carcinoma. Asian Pac J Cancer Prev. 2013;14(6):3831-6.

101. Sobolewski C, Abegg D, Berthou F, Dolicka D, Calo N, Sempoux C, et al. S100A11/ANXA2 belongs to a tumour suppressor/oncogene network deregulated early with steatosis and involved in inflammation and hepatocellular carcinoma development. Gut. 2020;69(10):1841-54.102. Hsu SH, Wang LT, Lee KT, Chen YL, Liu KY, Suen JL, et al. Proinflammatory homeobox gene, ISX, regulates tumor growth and survival in hepatocellular carcinoma. Cancer Res. 2013;73(2):508-18.
103. Chuang KT, Wang SN, Hsu SH, Wang LT. Impact of bromodomain-containing protein 4 (BRD4) and intestine-specific homeobox (ISX) expression on the prognosis of patients with hepatocellular carcinoma' for better clarity. Cancer Med. 2021;10(16):5545-56.
104. Sun S, Xu MZ, Poon RT, Day PJ, Luk JM. Circulating Lamin B1 (LMNB1) biomarker detects early stages of liver cancer in patients. J Proteome Res. 2010;9(1):70-8.
105. Abdelghany AM, Rezk NS, Osman MM, Hamid AI, Al-Breedy AM, Abdelsattar HA. Using Lamin B1 mRNA for the early diagnosis of hepatocellular carcinoma: a cross-sectional diagnostic accuracy study. F1000Res. 2018;7:1339.
106. Yang Y, Gao L, Chen J, Xiao W, Liu R, Kan H. Lamin B1 is a potential therapeutic target and prognostic biomarker for hepatocellular carcinoma. Bioengineered. 2022;13(4):9211-31.
107. Martinez-Quetglas I, Pinyol R, Dauch D, Torrecilla S, Tovar V, Moeini A, et al. IGF2 Is Up-regulated by Epigenetic Mechanisms in Hepatocellular Carcinomas and Is an Actionable Oncogene Product in Experimental Models. Gastroenterology. 2016;151(6):1192-205.
108. Yang DY, Rogler CE. Analysis of insulin-like growth factor II (IGF-II) expression in neoplastic nodules and hepatocellular carcinomas of woodchucks utilizing in situ hybridization and immunocytochemistry. Carcinogenesis. 1991;12(10):1893-901.
109. Wang Z, Ruan YB, Guan Y, Liu SH. Expression of IGF-II in early experimental hepatocellular carcinomas and its significance in early diagnosis. World J Gastroenterol. 2003;9(2):267-70.
110. Zhou Q, Mao YQ, Jiang WD, Chen YR, Huang RY, Zhou XB, et al. Development of IGF signaling antibody arrays for the identification of hepatocellular carcinoma biomarkers. PLoS One. 2012;7(10):e46851.
111. Tsai JF, Jeng JE, Chuang LY, You HL, Wang LY, Hsieh MY, et al. Serum insulin-like growth factor-II as a serologic marker of small hepatocellular carcinoma. Scand J Gastroenterol. 2005;40(1):68-75.
112. Chun YS, Huang M, Rink L, Von Mehren M. Expression levels of insulin-like growth factors and receptors in hepatocellular carcinoma: a retrospective study. World J Surg Oncol. 2014;12:231.
113. Zhang Y, Wang Y, Wei Y, Wu J, Zhang P, Shen S, et al. Molecular chaperone CCT3 supports proper mitotic progression and cell proliferation in hepatocellular carcinoma cells. Cancer Lett. 2016;372(1):101-9.
114. Hou JY, Wu HY, He RQ, Lin P, Dang YW, Chen G. Clinical and prognostic value of chaperonin containing T-complex 1 subunit 3 in hepatocellular carcinoma: A Study based on microarray and RNA-sequencing with 4272 cases. Pathol Res Pract. 2019;215(1):177-94.
115. Cui X, Hu ZP, Li Z, Gao PJ, Zhu JY. Overexpression of chaperonin containing TCP1, subunit 3 predicts poor prognosis in hepatocellular carcinoma. World J Gastroenterol. 2015;21(28):8588-604.
116. Zhang Z, Xu L, Sun C. Comprehensive characterization of cancer genes in hepatocellular carcinoma genomes. Oncol Lett. 2018;15(2):1503-10.
117. Iwako H, Tashiro H, Amano H, Tanimoto Y, Oshita A, Kobayashi T, et al. Prognostic significance of antithrombin III levels for outcomes in patients with hepatocellular carcinoma after curative hepatectomy. Ann Surg Oncol. 2012;19(9):2888-96.
118. Mizuguchi T, Kawamoto M, Meguro M, Son S, Nakamura Y, Harada K, et al. Serum antithrombin III level is well correlated with multiple indicators for assessment of liver function and diagnostic accuracy for predicting postoperative liver failure in hepatocellular carcinoma patients. Hepatogastroenterology. 2012;59(114):551-7.
119. Gao L, Ge C, Fang T, Zhao F, Chen T, Yao M, et al. ANGPTL2 promotes tumor metastasis in hepatocellular carcinoma. J Gastroenterol Hepatol. 2015;30(2):396-404.
120. Zhou J, Yang W, Zhang S, He X, Lin J, Zhou T, et al. Diagnostic value of angiopoietin-like protein 2 for CHB-related hepatocellular carcinoma. Cancer Manag Res. 2019;11:7159-69.
121. Bai Y, Lu D, Qu D, Li Y, Zhao N, Cui G, et al. The Role of ANGPTL Gene Family Members in Hepatocellular Carcinoma. Dis Markers. 2022;2022:1844352.

122. Li W, Miao X, Qi Z, Zeng W, Liang J, Liang Z. Hepatitis B virus X protein upregulates HSP90alpha expression via activation of c-Myc in human hepatocarcinoma cell line, HepG2. Virol J. 2010;7:45.
123. Wei W, Liu M, Ning S, Wei J, Zhong J, Li J, et al. Diagnostic value of plasma HSP90alpha levels for detection of hepatocellular carcinoma. BMC Cancer. 2020;20(1):6.
124. Zimmers TA, Jin X, Gutierrez JC, Acosta C, McKillop IH, Pierce RH, et al. Effect of in vivo loss of GDF-15 on hepatocellular carcinogenesis. J Cancer Res Clin Oncol. 2008;134(7):753-9.
125. Chen J, Tang D, Xu C, Niu Z, Li H, Li Y, et al. Evaluation of Serum GDF15, AFP, and PIVKA-II as Diagnostic Markers for HBV-Associated Hepatocellular Carcinoma. Lab Med. 2021;52(4):381-9.
126. Liu X, Chi X, Gong Q, Gao L, Niu Y, Chi X, et al. Association of serum level of growth differentiation factor 15 with liver cirrhosis and hepatocellular carcinoma. PLoS One. 2015;10(5):e0127518.
127. Myojin Y, Hikita H, Tahata Y, Doi A, Kato S, Sasaki Y, et al. Serum growth differentiation factor 15 predicts hepatocellular carcinoma occurrence after hepatitis C virus elimination. Aliment Pharmacol Ther. 2022;55(4):422-33.
128. Zhu C, Luo X, Wu J, et al. TM4SF1, a binding protein of DVL2 in hepatocellular carcinoma, positively regulates beta-catenin/TCF signalling. J Cell Mol Med. 2021;25(5):2356-2364.
129. Du X, Fan W, Chen Y. microRNA-520f inhibits hepatocellular carcinoma cell proliferation and invasion by targeting TM4SF1. Gene. 2018;657:30-38.
130. Lee SA, Lee SY, Cho IH, et al. Tetraspanin TM4SF5 mediates loss of contact inhibition through epithelial-mesenchymal transition in human hepatocarcinoma. J Clin Invest. 2008;118(4):1354-1366.
131. Sun H, Kim E, Ryu J, et al. TM4SF5-mediated liver malignancy involves NK cell exhaustion-like phenotypes. Cell Mol Life Sci. 2021;79(1):49.
132. Jung JW, Macalino SJY, Cui M, et al. Transmembrane 4 L Six Family Member 5 Senses Arginine for mTORC1 Signaling. Cell Metab. 2019;29(6):1306-1319.e7.
133. Feder S, Haberl EM, Spirk M, Weiss TS, Wiest R, Buechler C. Pentraxin-3 is not related to disease severity in cirrhosis and hepatocellular carcinoma patients. Clin Exp Med. 2020;20(2):289-97.
134. Deng H, Fan X, Wang X, Zeng L, Zhang K, Zhang X, et al. Serum pentraxin 3 as a biomarker of hepatocellular carcinoma in chronic hepatitis B virus infection. Sci Rep. 2020;10(1):20276.
135. Matsubara T, Kanto T, Kuroda S, Yoshio S, Higashitani K, Kakita N, et al. TIE2-expressing monocytes as a diagnostic marker for hepatocellular carcinoma correlates with angiogenesis. Hepatology. 2013;57(4):1416-25.
136. Mao L, Wang Y, Wang D, Han G, Fu S, Wang J. TEMs but not DKK1 could serve as complementary biomarkers for AFP in diagnosing AFP-negative hepatocellular carcinoma. PLoS One. 2017;12(9):e0183880.
137. Wight TN, Kang I, Evanko SP, Harten IA, Chang MY, Pearce OMT, et al. Versican-A Critical Extracellular Matrix Regulator of Immunity and Inflammation. Front Immunol. 2020;11:512.
138. Xia L, Huang W, Tian D, Zhang L, Qi X, Chen Z, et al. Forkhead box Q1 promotes hepatocellular carcinoma metastasis by transactivating ZEB2 and VersicanV1 expression. Hepatology. 2014;59(3):958-73.
139. Zhangyuan G, Wang F, Zhang H, Jiang R, Tao X, Yu D, et al. VersicanV1 promotes proliferation and metastasis of hepatocellular carcinoma through the activation of EGFR-PI3K-AKT pathway. Oncogene. 2020;39(6):1213-30.
140. Li Z, Qu L, Luo W, Tian Y, Zhai H, Xu K, et al. Mig-6 is down-regulated in HCC and inhibits the proliferation of HCC cells via the P-ERK/Cyclin D1 pathway. Exp Mol Pathol. 2017;102(3):492-9.
141. Ezzikouri S, Kimura K, Sunagozaka H, Kaneko S, Inoue K, Nishimura T, et al. Serum DHCR24 Auto-antibody as a new Biomarker for Progression of Hepatitis C. EBioMedicine. 2015;2(6):604-12.
142. Wu Y, Min J, Ge C, et al. Interleukin 22 in Liver Injury, Inflammation and Cancer. Int J Biol Sci. 2020;16(13):2405-2413.
143. Zeng T, Guan Y, Li YK, Wu Q, Tang XJ, Zeng X, et al. The DNA replication regulator MCM6: An emerging cancer biomarker and target. Clin Chim Acta. 2021;517:92-8.
144. Liu Z, Li J, Chen J, Shan Q, Dai H, Xie H, et al. MCM family in HCC: MCM6 indicates adverse tumor features and poor outcomes and promotes S/G2 cell cycle progression. BMC Cancer. 2018;18(1):200.
145. Liao X, Liu X, Yang C, Wang X, Yu T, Han C, et al. Distinct Diagnostic and Prognostic Values of Minichromosome Maintenance Gene Expression in Patients with Hepatocellular Carcinoma. J Cancer. 2018;9(13):2357-73.

146. Jia W, Xie L, Wang X, Zhang Q, Wei B, Li H, et al. The impact of MCM6 on hepatocellular carcinoma in a Southern Chinese Zhuang population. Biomed Pharmacother. 2020;127:110171.
147. Liu M, Hu Q, Tu M, Wang X, Yang Z, Yang G, et al. MCM6 promotes metastasis of hepatocellular carcinoma via MEK/ERK pathway and serves as a novel serum biomarker for early recurrence. J Exp Clin Cancer Res. 2018;37(1):10.
148. Berasain C, Castillo J, Perugorria MJ, Prieto J, Avila MA. Amphiregulin: a new growth factor in hepatocarcinogenesis. Cancer Lett. 2007;254(1):30-41.
149. Awad AE, Ebrahim MA, Eissa LA, El-Shishtawy MM. Dickkopf-1 and Amphiregulin as Novel Biomarkers and Potential Therapeutic Targets in Hepatocellular Carcinoma. International Journal of Hematology-Oncology and Stem Cell Research. 2019.
150. Kang X, Sun L, Guo K, Shu H, Yao J, Qin X, et al. Serum protein biomarkers screening in HCC patients with liver cirrhosis by ICAT-LC-MS/MS. J Cancer Res Clin Oncol. 2010;136(8):1151-9.
151. Omran MM, Emran TM, Farid K, Eltaweel FM, Omar MA, Bazeed FB. An Easy and Useful Noninvasive Score Based on alpha-1-acid Glycoprotein and C-Reactive Protein for Diagnosis of Patients with Hepatocellular Carcinoma Associated with Hepatitis C Virus Infection. J Immunoassay Immunochem. 2016;37(3):273-88.
152. Astrom E, Stal P, Zenlander R, Edenvik P, Alexandersson C, Haglund M, et al. Reverse lectin ELISA for detecting fucosylated forms of alpha1-acid glycoprotein associated with hepatocellular carcinoma. PLoS One. 2017;12(3):e0173897.
153. Zhang G, Ha SA, Kim HK, Yoo J, Kim S, Lee YS, et al. Combined analysis of AFP and HCCR-1 as an useful serological marker for small hepatocellular carcinoma: a prospective cohort study. Dis Markers. 2012;32(4):265-71.
154. Zhu R, Xiao J, Luo D, Dong M, Sun T, Jin J. Serum AKR1B10 predicts the risk of hepatocellular carcinoma - A retrospective single-center study. Gastroenterol Hepatol. 2019;42(10):614-21.
155. Han C, Gao L, Bai H, Dou X. Identification of a role for serum aldo-keto reductase family 1 member B10 in early detection of hepatocellular carcinoma. Oncol Lett. 2018;16(6):7123-30.
156. Cheng K, Shi J, Liu Z, Jia Y, Qin Q, Zhang H, et al. A panel of five plasma proteins for the early diagnosis of hepatitis B virus-related hepatocellular carcinoma in individuals at risk. EBioMedicine. 2020;52:102638.
157. Hong L, Zhou Y, Xie X, Wu W, Shi C, Lin H, et al. A stemness-based eleven-gene signature correlates with the clinical outcome of hepatocellular carcinoma. BMC Cancer. 2021;21(1):716.
158. Song D, Zhou Z, Zhang D, Wu J, Hao Q, Zhao L, et al. Identification of an Endoplasmic Reticulum Stress-Related Gene Signature to Evaluate the Immune Status and Predict the Prognosis of Hepatocellular Carcinoma. Front Genet. 2022;13:850200.
159. Hassani SF, Sayaf M, Danandeh SS, Nourollahzadeh Z, Shahmohammadi M, Akbari S, et al. Novel Insight Into the Association Between Obesity and Hepatocellular Carcinoma Occurrence and Recurrence: High-Throughput Microarray Data Set Analysis of Differentially Expressed Genes. JCO Clin Cancer Inform. 2021;5:1169-80.
160. Wu C, Luo Y, Chen Y, Qu H, Zheng L, Yao J. Development of a prognostic gene signature for hepatocellular carcinoma. Cancer Treat Res Commun. 2022;31:100511.
161. Alzamzamy A, Elsayed H, Abd Elraouf M, Eltoukhy H, Megahed T, Aboubakr A. Serum vascular endothelial growth factor as a tumor marker for hepatocellular carcinoma in hepatitis C virus-related cirrhotic patients. World J Gastrointest Oncol. 2021;13(6):600-11.
